# Survivor-driven development of a PROM for use in routine colorectal cancer care

**DOI:** 10.2340/1651-226X.2025.42032

**Published:** 2025-03-27

**Authors:** Johanne D Lyhne, Lise Gade, Laila Hansen, Anne Johansen, Allan ’Ben’ Smith, Lars Henrik Jensen, Lise Ventzel

**Affiliations:** a Department of Oncology, University Hospital of Southern Denmark – Vejle, Denmark; b Institute of Regional Health Research, Faculty of Health Sciences, University of Southern Denmark, Odense, Syddanmark, Denmark; c Survivor Representative, colorectal cancer survivor, Denmark; d Municipality of Vejle, , Vejle, Denmark; e The Daffodil Centre, The University of Sydney, A Joint Venture with Cancer Council NSW, Sydney, NSW; f Danish Colorectal Cancer Center South, Vejle Hospital - University Hospital of Southern Denmark, Vejle, Denmark

**Keywords:** Late effects, unmet needs, PRO, screening, patient and public involvement, patient engagement, implementation, barriers

## Abstract

**Background and purpose:**

Despite the availability of patient reported outcome (PRO) measures (PROMs) for assessing survivorship care needs, their successful implementation remains limited. This study aimed to improve the likelihood of implementation success by actively engaging end-users in developing a PROM designed to address implementation barriers.

**Patients and methods:**

Selected barriers for implementation were: (1) PROMs do not adequately address relevant issues, (2) PROMs can inhibit patient-clinician interaction, and (3) PROMs are not suitable for all patients. Management of these barriers were discussed at two 1-day workshops at Vejle Hospital with in-person attendance by colorectal cancer (CRC) survivors and informal caregivers (ICs). Relevant issues of CRC survivorship care (barrier 1) were defined based on data from four distinct sources. Solutions to overcoming barriers 2 and 3 were discussed at the workshops. Workshop data were guided by the Qualitative Analysis Guide of Leuven (QUAGOL) guide.

**Results:**

The four distinct sources provided data from 4,545 CRC survivors. Thirteen individuals attended the in-person workshops. The following constructs were identified as relevant (barrier 1): self-rated well-being relative to pre-diagnosis, late effects encompassing both psychological and physical aspects, the role of caregivers, identity considerations, support systems, economic impacts, rehabilitation needs, and information provision. Specific element (e.g., keywords, prioritisation and agenda-setting) were incorporated to facilitate patient-clinician interactions (barrier 2). All constructs were considered relevant across all stages of CRC survivorship (barrier 3). The final PROM comprised 34 items.

**Interpretation:**

This dialogue-tool is designed to address implementation barriers by providing direct feedback on relevant late effects and supportive care needs from CRC survivors to clinicians.

## Introduction

During the last decades advancements in cancer treatment have resulted in higher survival rates. This situation has created a population of *cancer survivors*, defined by the National Cancer Institute as ranging from cancer free survivors to cancer survivors with metastatic cancer who switch between periods with antineoplastic treatment and periods without treatment. Many face challenges returning to normal functioning due to significant physical, psychological or socio-economic late effects, and supportive care needs resulting in a reduced quality of life (QoL) [1–4]. These challenges are frequent [5], and often overlooked [6] despite recommendations for dedicated interventions preventing both the occurrence and the severity of late effects and supportive care needs in survivorship guidelines [7–9].

Challenges addressing survivorship problems in clinical practice persists, despite the availability of effective interventions for many issues. This highlights the need to adapt the current model of *cancer care*, which primarily emphasises the clinician’s perspective on disease control and current toxicity into the broader framework of *survivorship care.* As defined by the European Society for Medical Oncology (ESMO) [7], the Multinational Association of Supportive Care in Cancer (MASCC), and the American Society of Clinical Oncology (ASCO) [8], survivorship care encompasses addressing the comprehensive needs of cancer survivors throughout all stages of the cancer trajectory, from diagnosis to end of life. These needs span physical, psychological, health care systemic, informational, financial, fertility-related, sexual, spiritual, and relational dimensions [8].

Patient Reported Outcomes (PROs) can be integrated to ‘screen for and routinely evaluate’ late effects and survivorship care needs of cancer survivors [7, 8, 10, 11]. If successfully implemented, PROs may facilitate active patient involvement, foster communication between patients and clinicians, and facilitate delivery of supportive care, which might lead to improved symptom control, increased patient satisfaction, and improved QoL [10–14]. However, successful implementation of PROs in clinical practice continues to face challenges [15–17], especially if the PROs do not adequately address relevant issues, inhibit patient-clinician interaction, are not suitable for all patients, face technical difficulties in administration, or lack clinician feedback [12, 13, 18, 19].

While several PRO measures (PROMs) and item libraries assessing unmet needs and QoL-related domains [20–24] have been developed and validated, the purpose of this study was to addresses certain patient-related barriers associated with PROMs implementation by actively engaging end-users in the development process. The goal of the PROM is to serve as a dialogue tool informing clinical decision-making on relevant disease-specific late effects and survivorship care needs for colorectal cancer (CRC) survivors at all stages.

## Methods

This article focusses on three barriers to the successful implementation of PROMs that are directly related to the PROM content: (1) PROMs do not adequately address relevant issues, (2) PROMs can inhibit patient-clinician interaction, and (3) PROMs are not suitable for all patients. To address these barriers, we engaged the end-users of the PROM to guide us on content and formulation, and a Partner Panel of CRC survivors and informal caregivers (ICs) was established. The process of involving CRC survivors and ICs as research partners was guided by the Patient-Centered Outcomes Research Institute’s (PCORI) Foundational Expectations for Partnerships in Research [25]. The recommendations concerning the PRO respondent burden [26] were endorsed by survivors and ICs. Identification of the core constructs aligned with the Core Outcome Set-STAndards for Development (COS-STAD) recommendations [27]

The six foundational expectations from PCORI were addressed as follows:

1. Diversity & Representation

The target population for the PROM comprised survivors with various stages of CRC and ICs – together defined as the *Partner Panel*. We employed a multifaceted recruitment strategy with advertisements on Facebook, in the Danish Bowel Cancer Association (a patient organisation) newsletter, and oral invitations in the Department of Oncology, University Hospital of Southern Denmark – Vejle. Utilising purposive sampling, we recruited a diverse cohort, considering factors such as age, gender, cancer status, late effects, and experience with patient advocacy. The goal was to recruit 12–16 participants, ensuring a balance between survivors with cancer, cancer free survivors and ICs. Informed consent was obtained from all participants involved, and the study received approval from the Regional Ethical Committee at the University of Southern Denmark (Case no. 24/2931).

2. Early & Ongoing Engagement.

The Partner Panel was engaged in defining adequate CRC care, establishing core constructs for CRC care, and developing an aligning PROM. The Partner Panel also engaged in testing the PROM, refining the PROM according to feedback, planning implementation evaluation, and disseminating results.

3. Dedicated Funds for Engagement & Partner Compensation

The Partner Panel was compensated non-financially with catering during workshops. Parking was free of charge. No compensation was offered for transportation or time.

4. Build Capacity to Work as a Team

Workshops were scheduled at times and locations convenient for the Partner Panel, and were designed with a variety of activities in series of steps tailored to enhance equity in participation.

5. Meaningful Inclusion of Partners in Decision Making

Final decisions were made collaboratively. To be considered relevant, a symptom needed to have a minimum prevalence of 10% in the initial surveys, and a consensus threshold of 90% agreement among the Partner Panel was required to define a symptom as a core construct. Item formulation was achieved through joint discussion. In instances of disagreement, simple voting was employed.

6. Ongoing Review & Assessment of Engagement

Feedback was obtained at the end of each workshop. Engagement-related issues and concerns, such as those regarding privacy or confidentiality, were addressed in a respectful manner.

### Data sources

To identify the most important core constructs of CRC survivorship care, information on late effects and needs was gathered through various means:

1. Surveying CRC survivors undergoing active treatment regarding persisting side-effects (Additional file 1). November 2018 to March 2024.
2. Surveying a nationwide population of CRC survivors 5 to 10 years after diagnosis regarding late effects (Additional file 2). May 2023 to March 2024.
3. Posting queries on the ‘Late effects after colorectal cancer – LARS’ Facebook group (Additional file 3). February 2024 to March 2024.
4. Online meeting (1.5 h) with Danish Bowel Cancer Association volunteers (Additional file 4) discussing constructs related to colorectal cancer care. February 20, 2024.

The prevalence of each reported symptom from sources 1 and 2 was calculated based on the number of symptoms reported with moderate to severe severity. Qualitative analysis was conducted on the content from sources 3 and 4 to identify themes related to needs. The results were used as the basis for the workshops.

### Home assignments

Prior to the workshops, the Partner Panel completed a pre-assignment containing open-ended questions (Additional file 5). This survey enabled gathering of individual data regarding each member’s perspectives on the questions, free from the potential influence of group dynamics during the workshop.

### The workshops

The Partner Panel participated in two 1-day multi-method workshops with in-person attendance. The workshops encompassed various activities including plenary sessions outlining the purpose and the benefits of using PROMs in clinical practice, group discussions, and voting (agendas in additional file 6). The primary objective was to ensure engagement of all participants as equal partners in the process revolving around the questions listed in Table 1. Group work was facilitated by three unrelated moderators (two CRC physicians and one cancer rehabilitation nurse from the municipality of Vejle) with experience in quality improvement and research. All group moderators were prepared for the workshop during working meetings to ensure shared understanding of the aims and methods for each session and the overall study.

**Table 1:** Questions guiding the identification of CRC core outcome constructs.

1.	How do CRC survivors and their ICs experience CRC impact on their lives?
2.	How do CRC survivors and their ICs describe the most important constructs for survivorship care for CRC survivors?
3.	How do CRC survivors and their ICs suggest or believe these constructs could be measured?

CRC: colorectal cancer.

### Drafting the new Patient Reported Outcome Measure

Version 1 of the new PROM was developed based on the items formulated in response to question 3 (Table 1), supplemented by items from existing PROMs [20–24], which were closely examined during workshops. Where applicable, both the original wording and response scales were adopted directly; alternatively, the wording was modified to better reflect the specific construct of interest. A recognisable 4-point Likert-like scale (Not at all / A Little / Quite a Bit / Very Much), commonly used in PROMs (e.g. EORTCs), served as the basis for the response format. Less relevant items were excluded to adhere to the 10-min time cap for completing the measure set by the Partner Panel. Finally, additional items covering defined core constructs of CRC survivorship care not discussed during workshop due to limited time were crafted by the research team grounded in the qualitative data from the workshops (pre-assignments, post-it notes, session notes, flip-charts). The data were analysed through an iterative process guided by the Qualitative Analysis Guide of Leuven (QUAGOL) guide [28].

### Internal feedback of the new Patient Reported Outcome Measure

Version 1 of the new PROM was distributed to the Partner Panel for review. Open-ended qualitative feedback was gathered individually by e-mail, and amendments were made accordingly.

### External testing the new PROM

The external testing involved a separate cohort of volunteers from the Danish Bowel Cancer Association, comparable to end-users, to evaluate the applicability and feasibility of the proposed PROM. This group received no prior information about the core constructs the PROM aimed to measure. Participants were prompted to address specific questions (yes/no with the option to elaborate): (1) Are these items relevant to you? (2) Are the formulations acceptable and understandable? (3) Are the items relevant to your conversation with your oncologist? (4) Do you perceive any missing themes? Six external CRC survivors actively engaged in testing the new PROM. Their feedback was systematically collected and presented to the Partner Panel in workshop 2.

### Statistics

Descriptive measures were reported as absolute numbers with range for demographic measures. *Source 1 and 2:* Symptoms of anxiety, depression, and fear of cancer recurrence were dichotomised as ‘present’ or ‘not present’ based on established cut-offs [29, 30]. All physical symptoms were dichotomised based on severity with ‘not present’ corresponding to 0–2 on the Likert like response scale from 0 to 4 and ‘present’ corresponding to 3 or 4. In case of missing items within the PROMs, the item was replaced with a 0 indicating a conservative approach. If more than half of items were missing, the sum score could not be calculated. No imputation was performed.

## Results

The results from the four sources are listed in detail in supplementary 1–4. *Source 1*: PRO responses from 537 CRC survivors undergoing antineoplastic treatment were gathered. Mean respondent age was 63 years, and 36.5% were female. *Source 2*: Of the 6,989 surveyed, 3,955 (56.6%) responded. The mean age of respondents was 73 years, 41.5% were female. Average time since diagnosis was 7 years. *Source 3*: Forty-two responses were received from the Facebook group, with 75.6% of respondents being female. *Source 4*: Eleven participants (10 survivors and 1 caregiver) engaged in the virtual workshop. The mean age of participants was 65 years, and 91.7% were female. The average time since diagnosis was 4 years.

None of the symptoms (persistent nausea, diarrhoea, and neuropathy) from source 1 exceeded the established cut-off of 10%. Several symptoms from source 2 exceeded 10%, including bowel dysfunction (45.7%), fear of cancer recurrence (34.1%), tiredness (28.6%), sexual dysfunction (27.8%), urinary dysfunction (25.8%), cognitive dysfunction (19.3%), health anxiety (15.6%), neuropathy (12.4%), and dizziness (11.3%).

Further analysis showed that 74% of participants reporting elevated health anxiety also reported fear of cancer recurrence, and the isolated prevalence of health anxiety did not exceed 10%. The prevalence of anxiety and depression symptoms were 4.3% and 2.8%, respectively.

Data from source 3 and 4 revealed that late effects in general, how to get help for late effects, and the need for information regarding the cancer prognosis and trajectory were the most prevalent themes.

Clinical and demographic characteristics of the in-person workshop participants are listed in Table 2.

**Table 2:** Clinical and demographic characteristics of workshop participants.

Respondent type	Cancer-free CRC survivors	Advanced/metastatic CRC survivors	IC
Total	5	4	4
Age, years, mean (range)	68 (63–77)	55 (47–66)	69 (60–73)
Gender, female/male	4/1	2/2	2/2
**Diagnosis**			
Colon	2	3	N/A
Rectum	3	1	N/A
**Level of education**			
Low	2	0	1
Medium	2	3	2
High	1	1	1
**Employment status**			
In job	1	2	1
Not in job	4	2	3
Time since diagnosis, years, mean (range)	7.5 (2–12)	3.5 (>1–7.5)	N/A
Comorbidity (yes/no)	4/1	2/2	2/2
Quality of Life on a scale from 0–10, mean (range)	7.6 (4–10)	6 (4–9)	6.5 (4–10)
**Recruitment**			
Facebook	4	1	1
Another research project	1	2	0
Outpatient cancer clinic	0	1	0
As an informal caregiver	0	0	3
**Experience (more than 1 allowed)**			
Own experience	5	4	3
Representative from a society	0	1	1
Experience from previous research projects	1	2	0
Informal caregiver	0	0	3
**Region of cancer treatment**			
Region of Southern Denmark	3	1	1
Capital Region of Denmark	0	0	0
Region Zealand	0	1	0
Central Denmark Region	1	2	2
North Denmark Region	1	0	1
**Participating in**			
Workshop 1	4	3	3
Workshop 2	4	3	2

CRC: colorectal cancer; IC: informal caregiver.

### Workshop 1

*Question 1: How do CRC survivors and their IC experience CRC treatment impact on their lives?* The Partner Panel reported significant impacts of CRC on physical, psychological, and social functioning. The primary negative social effects were a restricted lifestyle due to bowel dysfunction, financial concerns, and role limitations. Fear of recurrence and disease progression were the most frequently reported psychological impacts. See Table 3 for an illustration of coding these experiences.

**Table 3:** Illustration of coding of experiences.

Outcome category	Example (individual)	Example (group)
**Physical impact**	‘I need to constantly think of what I eat. Fibers. Sugars. Coffee. Tomato. Just a little mistake and my stool is a total mess’. (Symptom distress – bowel dysfunction) ‘My feet are constantly tingling. It is a constant reminder of my cancer’. (Symptom distress – neuropathy)	‘There need to be a focus on sexual challenges from the health care staff. We don’t mention it, if we are not asked directly’. (Symptom distress – sexual dysfunction) ‘Nobody tells you what you may do or may not do physically. Can I run? Can I jump? How fast and how much?’ (Symptom distress – physical function )
**Psychological impact**	‘Knowing that the cancer can come back. That you never know for real, that you a cancer-free. It’s like a constant bag you have to carry. It tears me up’. (Psycho-oncological distress – worry about recurrence) ‘I never had any symptoms indicating cancer. It’s like my body just abandoned me. Sometimes I think the cancer is my own fault’. (Existential distress)	‘As a caregiver it could be nice with some kind of overview of what is about to happen and what can happen in the future. You might be the one who has to pass information on to the rest of the family’. (Distress of relatives – information) ‘You’ll never be who you were before. You need to start adapting to the new situation’. (Identical distress – role function) ‘You feel totally alone, even though your whole family is around you’. (Psychological distress – loneliness)
**Social impact**	‘I can’t have a normal job, because I spend all morning running back and forth to the toilet’. (Social impact – job function) ‘I won’t go swimming because of my stoma, and I’m always worried that it will be noisy or smelly when I’m out. So that happens rarely’. (Social impact – stoma distress)	‘It is a big part of life to always have to plan. You never have a day off. You mind is always somewhere else, and not with your family’ (Social impact – role function) ‘You don’t know what kind of job you can have in the future. Can you return to what you had or do you need to think about alternatives? The job is such a big part of your identity’. (Social impact – job function)

*Question 2: How do CRC survivors and their IC describe the most important constructs for survivorship care for CRC survivors?* Table 4 summarises the constructs identified as most important by the Partner Panel based on data from the four sources and research question 1. The overarching construct expressed by the Partner Panel was to accept the new life situation while feeling capable of managing the impact of cancer, including late effects.

**Table 4:** Outcomes of workshop 1 (core outcome constructs and items) with 10 participants.

Construct identified as important	Description of construct / underlying categories	Item formulated (version 1)
Well-being	Maintain physical health and functioning Exercise Walking the dog Doing grocery shopping ‘Doing a bit of what you did before’	**Have you started being physical active again?**
Physical	Get control of defecation Reduce pain/neuropathy Reduce frequency of urination	Are you experiencing bowel dysfunction? Are you experiencing stoma problems? Are you experiencing pain? Are you experiencing neuropathy? (Tingling or numbness in hands or feet?) Are you experiencing urinary dysfunction?
Sexual	Body image Sexual problems	Have you changed your perception of your body? Are you experiencing sexual challenges?
General	Extreme tiredness with multiple explanations Cognitive dysfunction	Are you experiencing difficulty sleeping? Have you felt tired? **Are you experiencing issues with memory, coordination, or planning?**
Psychological	Thoughts about recurrence and/or progression Change in mood and feelings Loneliness	Are you concerned or afraid that the cancer will return or progress? Have you been sad or upset? Do you tend to isolate yourself?
Existential	Change in beliefs and religion Existential thoughts Positive impact of cancer Post-traumatic growth	**Do you experience feelings of grief, shame, or guilt about your situation?** **Has your worldview changed since you were diagnosed with cancer?**
Economic	Clarity on legal rights Maintain job function Return to work	**Are you concerned about your finances?** **Have you given any thoughts to returning to work?**
Health care	Information on how diet affects the stool or stoma output Survivorship care / follow-up Secondary diseases Psycho-education What can I do myself to feel better? Do’s and don’ts	Do you have any questions regarding your diet? **Do you feel you have a good dialogue with your general practitioner?** **Have you considered pursuing alternative treatments alongside our treatment?**
Roles	Role functioning	**How do you function in your daily life compared to before you became ill – are your roles the same?**
Social	Relationship with partner and children Burden on relatives Openly talk about the important things and the difficult things with loved ones and health care professionals	**Does your current situation impact on your relatives to a degree where they require assistance?** Do you talk about the difficult things at home? **Do you find that there are important matters that are difficult to discuss with healthcare professionals?**
Information	Informational needs Support Community/peers	**Do you need more information about the course of cancer and life in the future?** Do you know where to seek help? Do you wish to get in contact with others in your situation?
Summarising	Quality of life Constructs not captured by the questionnaire Ranging constructs individually Preparation for the outpatient visit	**How is your quality of life compared to before you were diagnosed with cancer? (Excellent, good, the same, poor, very poor).** Are there other aspects that you believe are relevant but not included in this questionnaire? **Which of the above (symptoms, late effects, or other) impacts on your quality of life the most?** **Which of the above (symptoms, late effects, or other) would you like to discuss with healthcare professionals at your next appointment?**

Bolded text: Items formulated during workshop. Regular text: Items formulated by the research team based on constructs.

*Question 3: How do CRC survivors and their IC suggest or believe these constructs could be measured?* The Partner Panel responded positively to the aim of developing a digital tool for systematic reporting of PROM data as part of routine care. They emphasised the importance of including a final prioritisation step for the upcoming cancer care visit. The items directly formulated during the workshop are highlighted in bold in Table 4, located in the last column adjacent to the construct of interest. The items in regular text were compiled by the research team based on the qualitative data after workshop 1.

### Review of existing PROMs

Five existing validated PROMs were reviewed by the Partner Panel. The Cancer Survivors’ Unmet Needs (CaSUN) tool [20] did not capture the needs of survivors with advanced disease, and its response categories were found to be confusing. However, after reviewing the PROM, the Panel decided to include reworded items related to alternative medicine, peer support, body image, and post-traumatic growth. 9/42 items were deemed irrelevant.

The European Organization for Research and Treatment of Cancer (EORTC) Quality of Life Core [24] and ColoRectal cancer [23] questionnaires were too detailed to function effectively as dialogue tools. For instance, ‘urinary dysfunction’ was not addressed in a single item but rather uncovered with four different items on frequency during the day, frequency during the night, involuntary urination, and pain during urination. Participants did not consider detailed reporting on functional status (item 1–5) essential, as the new PROM was intended for use within in-person consultations with healthcare professionals. Items related to pain, difficulty sleeping, and fatigue were adopted directly. 12/30 items were deemed irrelevant. The EORTC questionnaire on palliation [22] was considered too generic, with six/15 items identified as irrelevant.

The Supportive Care Needs Survey (SCNS-34) [21] primarily focusses on healthcare services. 14/34 items were deemed irrelevant in a Danish health care setting and the remaining constructs were already covered.

### The new PROM, version 2

#### Results from workshop 2 and external testing

The outcomes of Workshop 2 are presented in Table 5 (regular text). Comments and suggestions from external testing are highlighted in bold. Only minor adjustments were made after internal testing (not depicted).

**Table 5:** Outcomes of workshop 2 with 9 participants (regular text).

Comment/suggestion:	Change/additions:
To illustrate it is not only about exercising, but also includes walking the dog, taking the trash out, vacuum cleaning etc.	New version: Have you started incorporating physical activity into your everyday life?
**Examples are added to understand the item correctly.**	New version: Are you experiencing any bowel dysfunction? (Diarrhoea, constipation, bloating, heaviness or pain?)
**The possibility to write down keywords is needed as this is very personal matter.**	New version: Are you experiencing any sexual challenges? Please write down keywords here so we can help you discuss them.
**Sexual problems are not only about sex.**	New item added: Are you interested in intimacy with your partner?
Tired is ‘normal’. Fatigue captures the construct better.	New version: Have you felt fatigued?
**To capture the fact that returning to work is recommended and expected (for cancer-free survivors), and for the item to be relevant for those who are self-employed and retired, the formulation is changed.**	New version: Do you need to discuss your employment situation?
Diet is also about appetite.	New version. Do you have any questions regarding your diet or appetite?
To illustrate the role of the municipalities regarding primary prevention, psycho-education, community.	New item added: Are you aware of the various services available in your municipality?
Not all have an ‘at home’ and not all wants to talk about the difficult thing.	New version: Do you discuss the thing you would like to with your loved ones?
**The possibility to write down keywords is needed. This will make it easier to initiate a conversation.**	New version: Do you find that there are important matters that are difficult to discuss with healthcare professionals? If yes: Please write down keywords here so we can help you discuss them.

Bolded text: Comments from external testing.

In addition to these adjustments, response categories were changed from graded scales to yes/no option where relevant and an introductory text was formulated to explain the purpose of the PROM. Of the 32 items initially formulated (Table 4), 8 were rephrased during the second workshop, and 2 additional items were added (Table 5). The final version of the PROM contained 17 items related to physical, psychological, and existential well-being with graded response options, 13 items related to social and informational well-being with yes/no response options, 1 item assessing global QoL, and 3 free-text items for summarising and prioritising issues for the upcoming cancer care visit. The final 34-item PROM, estimated by the Partner Panel to adhere to the 10-min time-cap, can be found in Additional file 7.

## Discussion

This study aimed to support a Partner Panel in developing a dialogue tool for use in routine CRC survivorship care to foster routine screening and evaluation of late effects and survivorship care needs among CRC survivors of all stages. The process was designed to maximise the likelihood of successful implementation into routine clinical practice as the items were formulated by survivors and ICs based on qualitative and quantitative responses from thousands of peers.

The new PROM has undergone an iterative process including external testing and the final version was approved by the Partner Panel on the 30^th^ of April 2024. The working title of the new PROM is FRAME-PRO-CR.

FRAME-PRO-CR encompasses core constructs as defined by the Partner Panel, which align with the dimensions found in other PROMs [31]. These constructs include physical, psychological, informational, social, activities of daily living, healthcare, spiritual, sexual, economic, and role-related dimensions. What sets FRAME-PRO-CR apart from other questionnaires is its simplicity, the patient-oriented formulation (e.g., ‘grief, shame or guilt’ instead of ‘spiritual needs’ or ‘memory, coordination, or planning’ instead of ‘cognition’), along with the option to write keywords about taboo topics, which was highly appreciated by participants. Furthermore, the questionnaire concludes with a summary that helps survivors prepare and prioritise, and set the agenda for their upcoming consultation.

Keywords, prioritisation, and agenda-setting are expected to enhance the inclusion of the CRC survivor’s and IC’s late effects and supportive care needs during the clinical consultation by promoting active patient involvement, fostering communication between patients and clinicians, and facilitating the delivery of supportive care. This approach may, in turn, lead to improved symptom control, increased patient satisfaction, and enhanced QoL. Keywords, prioritisation and agenda-setting can be integrated into existing PROMs to enhance their implementation and clinical utility. However, involving end-users in the formulation of each specific items ensures that questions are framed in a way that allows them to provide responses that truly reflect their experiences and preferences. It can be argued that existing PROMs (e.g., EORTCs, CaSUN, and SCNS-34) focus more on comprehensive coverage of all supportive care needs, rather than fostering dialogue between patients and clinicians regarding which are most important to patients. While these PROMs are well-suited, for example, for remote monitoring or clinical trials, they may be less appropriate for routine clinical care.

Anxiety and depression are reported as frequent late effects after CRC in numerous papers [32–35], and are addressed in all of the reviewed existing PROMs targeting supportive care needs and late effects [20–22, 24]. However, the nationwide survey of long-term CRC survivors indicated a low prevalence of anxiety and depression, and none of the virtual or in-person workshop participants mentioned either anxiety or depression. Conversely, fear of cancer recurrence and progression was frequently reported and of significant concern among participants, as were feelings of sadness and experiences of grief. These constructs may overlap with anxiety and depression, and ongoing evaluation will assess the choice of formulation. To remain true to the Partner Panel, the PROM was finalised in the form they crafted and approved it.

FRAME-PRO-CR is a disease-specific questionnaire. While we initially considered creating a generic questionnaire, the relevance of each item and the time spent completing the questionnaire were rated as very important in both the virtual and in-person workshops. Upon approval of the final version of FRAME-PRO-CR, it became evident that most items are generic, and only a few items (e.g. bowel dysfunction, stoma, neuropathy and urinary dysfunction [item 2, 3, 5 and 6]) will need to be adjusted to accommodate survivors with for example lung cancer or breast cancer. To support this adaptation process, diagnosis-specific Partner Panels will need to be established.

### Strengths and limitations

The primary strengths of this study are the comprehensive and systematic approach to facilitating the development of a PROM by a Partner Panel, and the use of multiple data sources. The use of existing literature also provided valuable insights and informed the development of the PROM. Another strength of this study is its contemporary relevance. The language used and the constructs explored are framed within the current cancer treatment and societal context, ensuring that the findings are aligned with present-day experiences and healthcare challenges. We deviated slightly from existing guidelines on PROM development by presenting existing PROMs after the end-users had defined core constructs, rather than before, as we did not want to predefine the discussion or limit the focus to adapting existing measures.

A limitation to this study is the potential for selection bias in participant recruitment. The data from the four sources were all digital, indicating that the participants were likely to have some degree of digital literacy. Greater diversity in education levels and digital literacy could have been beneficial. The study utilised purposive sampling to recruit a diverse cohort of CRC survivors and their informal caregivers; however, the participants may not fully represent the entire population of CRC survivors, which could limit the generalisability of the findings. In addition, most workshop participants were female, which may have influenced the formulation of certain items. However, as the constructs are based on a large and diverse dataset, it is unlikely that this gender imbalance significantly impacted the definition of the constructs on which items were based.

Furthermore, the study did not include a formal validation of the developed FRAME-PRO-CR, but the content and face validity were evaluated by the six external test-participants. Further studies should assess the psychometric properties and reliability of FRAME-PRO-CR and collect health professionals’ perspectives on implementation. Finally, process validation of continuous maintenance and revision of the tool is necessary to reflect new side effects and emerging needs, with special caution to the FRAME-PRO-CR’s ability to detect and address late effects and survivorship care needs for individuals approaching palliative stages.

### Clinical implications

This study contributes to the growing body of literature on PROMs in routine cancer care. By addressing some of the patient-related challenges associated with the implementation of PROMs (addresses relevant issues, facilitates patient-clinician interaction, is suitable for all survivors regardless of disease status), the FRAME-PRO-CR has the potential to improve the quality of CRC survivorship care by enabling more personalised care targeting the specific late effects and supportive care needs of each survivor *if* the PROM is successfully integrated into the existing workflow allowing for clinician feedback.

Future evaluation of the impact of implementing FRAME-PRO-CR will provide valuable insights into the effectiveness of this approach in improving survivorship care. Pilot testing is currently underway at the CRC unit within the Department of Oncology at Vejle Hospital. The results of this pilot will play a key role in refining the implementation process for use in related units. Scaling the approach to non-oncological departments managing cancer patients, such as medicine and surgery, will require prioritisation by stakeholders (e.g. hospital directors, policymakers, and chief physicians) and will depend on the outcomes of the initial implementation and its demonstrated impact on survivorship care for cancer patients. FRAME-PRO-CR has the potential to be implemented in health care settings across and beyond Denmark, but will require an adaptation process to suit local or national contexts.

## Conclusion

By involving CRC survivors and ICs in the rigorous development, FRAME-PRO-CR has been created. This dialogue-tool is designed to address patient-related implementation barriers by providing direct feedback on relevant late effects and supportive care needs from CRC survivors to clinicians. Key features of FRAME-PRO-CR include the formulation of items by CRC survivors and ICs, use of keywords, prioritisation, and agenda-setting. Future research will focus on the implementation process and assess the clinical impact of integrating FRAME-PRO-CR into routine clinical care.

## Author contributions

JDL: Study design, workshop facilitator, data collection, data analysis, interpretation of data and drafting manuscript. LG and LH: Workshop participants, interpretation of data and review of manuscript. LHJ: Concept, collection, interpretation of data and review of manuscript. ABS: Discussion of results and review of manuscript. LV: Workshop facilitator, data collection, discussion of results and review of manuscript. All authors have approved the final manuscript for publication.

## Supplementary Material

Survivor-driven development of a PROM for use in routine colorectal cancer care

## Data Availability

The dataset generated during this study is available from the corresponding author upon reasonable request.

## References

[1] Jefford M, Ward AC, Lisy K, Lacey K, Emery JD, Glaser AW, et al. Patient-reported outcomes in cancer survivors: a population-wide cross-sectional study. Support Care Cancer. 2017;25(10):3171–9. 10.1007/s00520-017-3725-5 28434095

[2] Simard S, Thewes B, Humphris G, Dixon M, Hayden C, Mireskandari S, et al. Fear of cancer recurrence in adult cancer survivors: a systematic review of quantitative studies. J Cancer Surviv. 2013;7(3):300–22. 10.1007/s11764-013-0272-z 23475398

[3] Glaser AW, Fraser LK, Corner J, Feltbower R, Morris EJ, Hartwell G, et al. Patient-reported outcomes of cancer survivors in England 1–5 years after diagnosis: a cross-sectional survey. BMJ Open. 2013;3(4): e002317. 10.1136/bmjopen-2012-002317 PMC3641492 23578682

[4] Downing A, Morris EJ, Richards M, Corner J, Wright P, Sebag-Montefiore D, et al. Health-related quality of life after colorectal cancer in England: a patient-reported outcomes study of individuals 12 to 36 months after diagnosis. J Clin Oncol. 2015;33(6):616–24. 10.1200/jco.2014.56.6539 25559806

[5] Sundhedsstyrelsen. Senfølger efter kræft hos voksne. [cited September 26th 2024] Available from: https://www.sst.dk/da/viden/sygdomme/kraeft/rehabilitering-og-palliation-paa-kraeftomraadet/senfoelger-efter-kraeft-hos-voksne

[6] Bekæmpelse K. Kræftpatienternes behov og oplevelse med sundhedsvæsenet i opfølgnings- og efterforløbet. Kræftens Bekæmpelses Barometerundersøgelse. 2019. [cited September 26th 2024] Available from: https://www.cancer.dk/dyn/resources/File/file/3/8373/1574778638/kraeftens-bekaempelses-barometerundersoegelse-2019.pdf

[7] Vaz-Luis I, Masiero M, Cavaletti G, Cervantes A, Chlebowski RT, Curigliano G, et al. ESMO Expert Consensus Statements on Cancer Survivorship: promoting high-quality survivorship care and research in Europe. Ann Oncol. 2022;33(11):1119–33. 10.1016/j.annonc.2022.07.1941 35963481

[8] Hart NH, Nekhlyudov L, Smith TJ, Yee J, Fitch MI, Crawford GB, et al. Survivorship care for people affected by advanced or metastatic cancer: MASCC-ASCO standards and practice recommendations. Support Care Cancer. 2024;32(5):313. 10.1007/s00520-024-08465-8 38679639 PMC11056340

[9] Sundhedsstyrelsen. Pakkeforløb for kræft i tyk- og endetarm. [cited September 26th 2024] Available from: https://www.sst.dk/da/udgivelser/2022/pakkeforloeb-for-kraeft-i-tyk-og-endetarm

[10] Graupner C, Kimman ML, Mul S, Slok AHM, Claessens D, Kleijnen J, et al. Patient outcomes, patient experiences and process indicators associated with the routine use of patient-reported outcome measures (PROMs) in cancer care: a systematic review. Support Care Cancer. 2021;29(2):573–93. 10.1007/s00520-020-05695-4 32875373 PMC7767901

[11] Di Maio M, Basch E, Denis F, Fallowfield LJ, Ganz PA, Howell D, et al. The role of patient-reported outcome measures in the continuum of cancer clinical care: ESMO Clinical Practice Guideline. Ann Oncol. 2022;33(9):878–92. 10.1016/j.annonc.2022.04.007 35462007

[12] Campbell R, Ju A, King MT, Rutherford C. Perceived benefits and limitations of using patient-reported outcome measures in clinical practice with individual patients: a systematic review of qualitative studies. Qual Life Res. 2022;31(6):1597–620. 10.1007/s11136-021-03003-z 34580822

[13] Yang LY, Manhas DS, Howard AF, Olson RA. Patient-reported outcome use in oncology: a systematic review of the impact on patient-clinician communication. Support Care Cancer. 2018;26(1):41–60. 10.1007/s00520-017-3865-7 28849277

[14] Licqurish SM, Cook OY, Pattuwage LP, Saunders C, Jefford M, Koczwara B, et al. Tools to facilitate communication during physician-patient consultations in cancer care: an overview of systematic reviews. CA Cancer J Clin. 2019;69(6):497–520. 10.3322/caac.21573 31339560

[15] Lai-Kwon J, Rutherford C, Jefford M, Gore C, Best S. Using implementation science frameworks to guide the use of electronic patient-reported outcome symptom monitoring in routine cancer care. JCO Oncol Pract. 2024;20(3):335-349. 10.1200/op.23.00462 38206290

[16] Roberts NA, Alexander K, Wyld D, Janda M. What is needed by staff to implement PROMs into routine oncology care? A qualitative study with the multi-disciplinary team. Eur J Cancer Care. 2019;28(6):e13167. 10.1111/ecc.13167 31603590

[17] Briggs MS, Rethman KK, Crookes J, Cheek F, Pottkotter K, McGrath S, et al. Implementing patient-reported outcome measures in outpatient rehabilitation settings: a systematic review of facilitators and barriers using the consolidated framework for implementation research. Arch Phys Med Rehabil. 2020;101(10):1796–812. 10.1016/j.apmr.2020.04.007 32416149

[18] Howell D, Molloy S, Wilkinson K, Green E, Orchard K, Wang K, et al. Patient-reported outcomes in routine cancer clinical practice: a scoping review of use, impact on health outcomes, and implementation factors. Ann Oncol. 2015;26(9):1846–58. 10.1093/annonc/mdv181 25888610

[19] Pappot H, Taarnhøj GA, Bentsen L, Friis RB, Bæksted C, Christiansen MG, et al. Patient-reported outcomes used actively in cancer patients undergoing antineoplastic treatment: a mini-review of the Danish landscape. Comput Struct Biotechnol J. 2024;24:23–30. 10.1016/j.csbj.2023.11.054 38076643 PMC10704259

[20] Hodgkinson K, Butow P, Hunt GE, Pendlebury S, Hobbs KM, Lo SK, et al. The development and evaluation of a measure to assess cancer survivors’ unmet supportive care needs: the CaSUN (Cancer Survivors’ Unmet Needs measure). Psycho-oncology. 2007;16(9):796–804. 10.1002/pon.1137 17177268

[21] Boyes A, Girgis A, Lecathelinais C. Brief assessment of adult cancer patients’ perceived needs: development and validation of the 34-item Supportive Care Needs Survey (SCNS-SF34). J Evaluation Clin Pract. 2009;15(4):602–6. 10.1111/j.1365-2753.2008.01057.x 19522727

[22] Groenvold M, Petersen MA, Aaronson NK, Arraras JI, Blazeby JM, Bottomley A, et al. The development of the EORTC QLQ-C15-PAL: a shortened questionnaire for cancer patients in palliative care. Eur J Cancer. 2006;42(1):55–64. 10.1016/j.ejca.2005.06.022 16162404

[23] Whistance R, Conroy T, Chie W, Costantini A, Sezer O, Koller M, et al. Clinical and psychometric validation of the EORTC QLQ-CR29 questionnaire module to assess health-related quality of life in patients with colorectal cancer. Eur J Cancer. 2009;45(17):3017–26. 19765978 10.1016/j.ejca.2009.08.014

[24] Aaronson NK, Ahmedzai S, Bergman B, Bullinger M, Cull A, Duez NJ, et al. The European Organization for Research and Treatment of Cancer QLQ-C30: a quality-of-life instrument for use in international clinical trials in oncology. J Natl Cancer Inst. 1993;85(5):365–76. 10.1093/jnci/85.5.365 8433390

[25] Institute P-COR. Engagement in research: foundational expectations for partnerships 2024. [cited September 26th 2024] Available from: https://www.pcori.org/sites/default/files/PCORI-Engagement-in-Research-Foundational-Expectations-for-Partnerships.pdf

[26] Aiyegbusi OL, Cruz Rivera S, Roydhouse J, Kamudoni P, Alder Y, Anderson N, et al. Recommendations to address respondent burden associated with patient-reported outcome assessment. Nat Med. 2024;30(3):650-659. 10.1038/s41591-024-02827-9 38424214

[27] Kirkham JJ, Davis K, Altman DG, Blazeby JM, Clarke M, Tunis S, et al. Core outcome Set-STAndards for development: the COS-STAD recommendations. PLoS Med. 2017;14(11):e1002447. 10.1371/journal.pmed.1002447 29145404 PMC5689835

[28] Dierckx de Casterlé B, Gastmans C, Bryon E, Denier Y. QUAGOL: a guide for qualitative data analysis. Int J Nurs Stud. 2012;49(3):360–71. 10.1016/j.ijnurstu.2011.09.012 21996649

[29] Simard S, Savard J. Fear of Cancer Recurrence Inventory: development and initial validation of a multidimensional measure of fear of cancer recurrence. Support Care Cancer. 2009;17(3):241–51. 10.1007/s00520-008-0444-y 18414902

[30] Christensen KS, Fink P, Toft T, Frostholm L, Ornbøl E, Olesen F. A brief case-finding questionnaire for common mental disorders: the CMDQ. Fam Pract. 2005;22(4):448–57. 10.1093/fampra/cmi025 15814580

[31] Rimmer B, Crowe L, Todd A, Sharp L. Assessing unmet needs in advanced cancer patients: a systematic review of the development, content, and quality of available instruments. J Cancer Surviv. 2022;16(5):960–75. 10.1007/s11764-021-01088-6 34363187 PMC9489568

[32] Aminisani N, Nikbakht H, Asghari Jafarabadi M, Shamshirgaran SM. Depression, anxiety, and health related quality of life among colorectal cancer survivors. J Gastrointest Oncol. 2017;8(1):81–8. 10.21037/jgo.2017.01.12 28280612 PMC5334052

[33] Brandenbarg D, Maass S, Geerse OP, Stegmann ME, Handberg C, Schroevers MJ, et al. A systematic review on the prevalence of symptoms of depression, anxiety and distress in long-term cancer survivors: implications for primary care. Eur J Cancer Care. 2019;28(3):e13086. 10.1111/ecc.13086 PMC9286037 31087398

[34] Mitchell AJ, Ferguson DW, Gill J, Paul J, Symonds P. Depression and anxiety in long-term cancer survivors compared with spouses and healthy controls: a systematic review and meta-analysis. Lancet Oncol. 2013;14(8):721–32. 10.1016/s1470-2045(13)70244-4 23759376

[35] Mols F, Schoormans D, de Hingh I, Oerlemans S, Husson O. Symptoms of anxiety and depression among colorectal cancer survivors from the population-based, longitudinal PROFILES Registry: prevalence, predictors, and impact on quality of life. Cancer. 2018;124(12):2621–8. 10.1002/cncr.31369 29624635 PMC6033166

