# Supplementary material for: Survivor-driven development of a PROM for use in routine colorectal cancer care
Source: Acta Oncol. 2025 Mar 27;64:42032. doi: 10.2340/1651-226X.2025.42032 (PMC11980947; doi:10.2340/1651-226X.2025.42032)
Supplement: Survivor-driven development of a PROM for use in routine colorectal cancer care [file AO-64-42032-s1.pdf]

Supplementary material has been published as submitted. It has not been copyedited, or typeset by Acta Oncologica

Additional file 1: Surveying colorectal cancer survivors undergoing active treatment

Additional file 2: Surveying a nationwide population of colorectal cancer survivors 5-10 years after diagnosis

Additional file 3: Queries posted on the Facebook wall of the group “Late effects after colorectal cancer – LARS”

Additional file 4: Online meeting with the Danish Bowel Cancer Association

Additional file 5: Home assignments prior to workshop 1

Additional file 6: Workshop agendas

Additional file 7: The final version of the FRAME-PRO-CR

Additional file 1. Colorectal cancer survivors undergoing active antineoplastic treatment. Surveyed prior to treatment regarding specific side-effects related to treatment toxicity.

	N = 537
Age in years, mean (range)	63 (37-85)
Sex, female	196 (36.5 %)
Nausea during treatment	77 (14.3 %)
Persistent nausea	47 (8.8 %)
Diarrhea during treatment	57 (10.6 %)
Persistent diarrhea	46 (8.6 %)
Tingling, numbness, or reduced sensitivity in the hands or feet	75 (14.0 %)
Persistent tingling in fingers/toes	51 (9.5 %)

Additional file 2. Cancer-free colorectal cancer survivors 5-10 years after diagnosis. National, cross-sectional survey on cancer related late-effects.

	N = 3,596		
Age in years, mean (range)	72.7 (30 – 96)		
Sex, female	1,493 (41,5 %)		
Time since diagnosis, mean, sd (range)	7.1, 1.4 (4.4 – 9.9)		
Cancer type			
Colon	2,375 (66 %)		
Rectum	1,221 (34 %)		
		Percentages	Missing
Physical symptoms within the last 4 weeks			
Nausea	228	6.4	52
Diarrhea	523	14.7	48
Other symptoms related to bowel dysfunction[1]	1,632	45.7	26
Neuropathy	439	12.4	56
Urinary dysfunction[2]	922	25.8	19
Sexual dysfunction	966	27.8	120
General symptoms within the last 4 weeks			
Tiredness	1,017	28.6	35
Headache	289	8.1	43
Cognitive dysfunction[3]	688	19.3	34
Dizziness	403	11.3	45
Psychological symptoms within the last 4 weeks			
Fear of cancer recurrence (FCRI-SF > / = 13)	1227	34.1	0 (mandatory)
Depression	99	2.8	117
Anxiety	153	4.3	18
Health anxiety	558	15.6	15
1: Loose stool, bowel pain, bloating, sensation of tension or heaviness in the abdomen, involuntary passing of gas and/or stool, difficulty emptying the bowel during defecation, or urge<br>2: Involuntary urination, bleeding from the bladder, frequent urination, difficulty emptying the bladder or bladder pain<br>3: Difficulty concentration or remembering			

Additional file 3. Queries posted on the Facebook wall of the group "Late effects after colorectal cancer – LARS"

Question 1: *What aspects do you wish you had discussed with your doctor during your cancer treatment?* (27 answers, 6 from male participants).

Themes (ordered according to frequency)

Late effects in general (21 statements)

How to receive help regarding late effects (10 statements)

Information regarding the cancer trajectory and future (9 statements)

Psychological late effects (2 statements)

Question 2: *What information do you wish you had prior to initiating your cancer treatment?* (8 answers, 3 from male participants).

Themes (ordered according to frequency)

Knowledge about what to expect during and after treatment (6 statements)

Information about where to seek help (3 statements)

Overview of the trajectory (2 statements)

Question 3: *If you were to put a measure on your quality of life today, what would make the most sense to you?* (7 answers, 2 from male participants).

Answers (ordered according to frequency)

Numbers from 1-10.

Colors: Red – yellow – green

Additional file 4. Online meeting with the Danish Bowel Cancer Association

12 participants, 11 women.

10 survivors, 2 informal caregivers.

Mean age 65 (range 50 – 79)

Mean time since diagnosis: 4 years (range 2-10)

Burden of late effects:

1-2 bothering late effects: 4

Many bothering late effects: 6

How long time would you accept spending on completing a questionnaire prior to your clinical appointment?

10-15 minutes (range 5 – 20)

1. "*What specific aspects do we absolutely need to include in our Patient-Reported Outcome Measures (PROM)?"* (20 responses)

Themes:

Comprehensive Understanding of Late Effects (15 statements)

Specific Concerns and Needs (5 statements)

Preparedness for Late Effects (3 statements)

2. "*What could have improved your cancer journey?"* (13 responses)

Themes:

Information and Follow-up (8 Statements)

Satisfaction and Adequacy of Care (5 Statements)

Coordination and Accessibility (4 Statements)

Mental Health Support (2 Statements)

Standardization of Services (2 Statements)

Additional file 5. Home assignments prior to workshop 1

Implementation of PROM in the Colorectal Cancer Department, Vejle Hospital

Pre-Workshop 1 Assignments:

1. The treatment for colorectal cancer affects my life physically in the following ways:

a. ____

b. ____

2. The treatment for colorectal cancer affects my life psychologically and/or socially in the following ways:

a. ____

b. ____

3. My greatest concern relates to: ____________________________________

4. The most important topics for me to discuss with my doctor (the one treating/treated my colorectal cancer) are:____

5. My treatment for colorectal cancer could have been better if:

____

6. Would you be willing to answer a questionnaire before each doctor's appointment? Yes / No

7. How many minutes would you be willing to spend on it?

Name:

Additional file 6. Workshop agendas

WORKSHOP 1

10:00 Welcome, presentation of facilitators

1st session.

Plenary Session: Aim, Purpose, and Methods

During the first session, the project's aim was introduced. The overarching goal was to enhance health and quality of life outcomes for CRC survivors and their IC by facilitating patient-driven development of an outcome measurement tool for use in routine outpatient cancer care, thereby ensuring a more personalized focus on individual needs and priorities.

2nd session, Focus Group, Part A

"Core Set of Person-Centered Colorectal Cancer Outcome Constructs to be Used in Routine Cancer Care."

Topic Coverage - What needs should be included in the PROM?

In the second session, three parallel moderated focus groups of one hour were conducted. The participants were randomly divided into three units. Participants shared their perspectives on how CRC had impacted on them, and their personal measures of successful cancer care. Data and quotes from peers gained through the source 3 and 4 were introduced at this level to initiate a discussion. The insights and discussions generated from each focus group session were systematically collected and summarized for further analysis.

Question 1: What should the consultation with the doctor in the outpatient clinic include besides "How are you?" or "Anything new since last time?"

Assisting Questions:

a. What would you like to discuss with the doctor during your cancer journey?

b. Were there any taboo topics or difficult subjects to address? Both as a survivor and as an informal caregiver.

c. Was there anything you felt the doctor should have addressed but didn't?

d. What is important to enhance the quality of overall treatment?

The moderator writes down on post-its everything you can think of.

3rd session, Exchange of post-its between groups. Categorization of post-its.

During the third session, a group work activity was conducted to prioritize and refine the compilation of the most important CRC outcomes and factors (including aspects related to care, support, and environment) essential for achieving successful care. Each group's findings were documented on flipcharts and notepads, and the outcomes were synthesized by the three group moderators into an overview. This overview served as the foundation for an open discussion, encouraging active participation from all attendees to reach consensus

on a comprehensive list of outcomes. Participants were involved in identifying which topics and constructs were applicable to all CRC survivors, and which were applicable only to a subgroup, if any. This process aimed to ensure inclusivity and capture the diverse needs and experiences within the CRC community.

Question 2: What is suitable for everyone, and what is suitable for selected groups? (Survivors with stomas, survivors with metastatic disease, younger survivors, etc.)
Presentation in groups, plenary discussion

12-12:45 Lunch (Facilitator: listing of topics)
4th session, Individual assessment
In the fourth session, a voting process was conducted, requiring each defined construct to achieve a minimum of 90% consensus to be considered highly important for inclusion in the core construct list.

5th session, Focus Group, Part B
In the fifth session, CRC survivors and their IC actively participated in discussions regarding the optimal way of measuring core constructs and the formulation of specific items. The aim was to ensure that the questions effectively captured the intended constructs of care. Groups undertook a meticulous examination of existing instruments (Supportive Care Needs Survey, SCNS-34 [21], Cancer Survivors' Unmet Needs (CaSUN) [20], and European Organization for Research and Treatment of Cancer (EORTC) core Quality of Life Questionnaire (QLQ-C30) [24], Colorectal (CR29) [23], and Palliative Care (PAL) [22], to ensure no core constructs were missed.
At the end of the meeting, participants were invited to share feedback on the process.
Question 3: How do we measure it? In other words, how should we ask? (based on a few specific topics)

Assisting Questions:
a. How do I ask you about this (diarrhea, fatigue, anxiety, or similar) so you have the opportunity to give me the most relevant answer?
b. How would you prefer to give your answer? Grading / numbers / colors?
Detailed questions or overarching with branching? Open questions?
c. How should we start? "Hello, how are you today?" "Anything new since last time?"
Coffee and cake
Wrap-up/Feedback of the day.
15:00 End of day

WORKSHOP 2

Group Work, Part 1. Three groups with facilitator.

10:00 Welcome, presentation of facilitators

1st session. Plenary Session: Aim, Purpose, and Methods

Content: Presentation of challenges in composing the PROM based on qualitative data extracted from workshop 1

Feedback from external testing

Purpose of the workshops

2nd session. Key Points. Finishing the new PROM

Key points:

a) Are all topics/areas included?

b) Are the formulations clear and concise?

c) Are (some of) the formulations too direct?

d) Are the formulations such that it makes sense to answer the questionnaire repeatedly?

e) Are the response categories satisfactory?

f) Is the order of questions optimal?

g) Should there be more "keywords" in some places?

h) Concerning work – should it be formulated differently?

i) Should nausea (8.8% during treatment, 6% 5-10 years after) and skin problems (12.5% during treatment) be included?

j) Should there be a division into physical quality of life and mental quality of life?

k) Should roles be divided into work and home?

l) Should "municipality" be mentioned anywhere? – In line with the general practitioner?

12 – 12.45: Lunch

3rd Session. Administration

The third session revolved around logistical planning, as groups collaboratively established protocols for the administration of the new PROM. Deliberations encompassed the timing of distribution, strategies for reminders, and contingency plans for reaching individuals unable to utilize electronic platforms, ensuring inclusivity and data security.

a) When should respondents answer? 10 days before? 7 days before? 3 days before? The day before? On the day?

b) What about reminders? When? How? How many?

c) What about those who do not respond electronically? Phone calls? Waiting room? Paper?

4th session. Evaluation.

In the fourth session, the groups engaged in discussions pertaining to the evaluation of the effects of implementing the new PROM within the CRC outpatient care setting. Group presentations followed to share insights and conclusions from the discussions. Finally, the workshop concluded with a summary and reflection on the day's proceedings.

a) What do we think the questionnaire will impact?

b) How should we evaluate the effect of using the questionnaire?

c) Should it be doctors, survivors, or relatives who evaluate?

d) Should it be interviews, or could some form of numerical data be envisaged?

Coffee and cake

Wrap-up/Feedback of the day.

15:00 End of day

Additional file 7: The final version of the PROM

How are you?
You have received this questionnaire because we would like to know how you are. Your answers will serve as a basis for our further conversation when we meet next time. We ask the same questions before each visit so we can help you in the best possible way and follow your progress during your time with us.

Before you answer, please note that:

- The information is considered part of your medical record, and only healthcare professionals involved in your treatment have access to it.
- Your answers may be used in an anonymized form for quality assurance and development in the healthcare system.

Some of the questions may be difficult to answer, but please try to answer as accurately as you can based on how you are at the moment. There are no right or wrong answers.
Please also indicate if a question is not relevant to you. If you require urgent care, please contact your department directly.

How are you - compared to before you had cancer?
(response options: not at all – a little – quite a bit – very much – not relevant)
1. Have you started incorporating physical activity into your everyday life?
2. Are you experiencing bowel dysfunction? (diarrhea, constipation, bloating, heaviness or pain)
3. Are you experiencing stoma problems?
4. Are you experiencing pain?
5. Are you experiencing neuropathy? (tingling or numbness in hands or feet?)
6. Are you experiencing urinary dysfunction?
7. Have you changed your perception of your body?
8. Are you interested in intimacy with your partner?
9. Are you experiencing any sexual challenges?
Please write any keywords here so we can help you discuss them: __________________
10. Are you experiencing difficulty sleeping?
11. Have you felt fatigued?
12. Are you experiencing issues with memory, coordination, or planning?
13. Are you concerned or afraid that the cancer will return or progress?
14. Have you been sad or upset?
15. Do you tend to isolate yourself?
16. Do you experience feelings of grief, shame, or guilt about your situation?
17. Has your worldview changed since you were diagnosed with cancer?

Questions about your surroundings (response options: yes – no)

18. Are you concerned about your finances?
19. Do you need to discuss your employment situation?
20. Do you have questions regarding your diet or appetite?
21. Do you feel you have a good dialogue with your general practitioner?
22. Are you aware of the various services available in your municipality?
23. Have you considered pursuing alternative treatments alongside our treatment?
24. How do you function in your daily life compared to before you became ill - are your roles the same?
25. Does your current situation impact on your relatives to a degree where they require assistance?
26. Do you discuss the thing you would like to with your loved ones?
27. Do you find that there are important matters that are difficult to discuss with healthcare professionals?

If yes: Please write any keywords here so we can help you discuss them: ___________________

28. Do you need more information about the course of cancer and life in the future?
29. Do you know where to seek help?
30. Do you wish to get in contact with others in your situation?

Summarizing/prioritizing

31. How is your quality of life compared to before you were diagnosed with cancer?

Response options: Excellent, good, the same, poor, very poor.

32. Are there other aspects that you believe are relevant but not included in this questionnaire?

____

33. Which of the above (symptoms, late effects, or other) impacts on your quality of life the most?____
34. Which of the above (symptoms, late effects, or other) would you like to discuss with healthcare professionals at your next appointment?

____
